# Amygdala and cingulate structure is associated with stereotype on sex-role

**DOI:** 10.1038/srep14220

**Published:** 2015-09-30

**Authors:** Hikaru Takeuchi, Yasuyuki Taki, Atsushi Sekiguchi, Rui Nouchi, Yuka Kotozaki, Seishu Nakagawa, Carlos Makoto Miyauchi, Kunio Iizuka, Ryoichi Yokoyama, Takamitsu Shinada, Yuki Yamamoto, Sugiko Hanawa, Tsuyoshi Araki, Hiroshi Hashizume, Keiko Kunitoki, Yuko Sassa, Ryuta Kawashima

**Affiliations:** 1 Division of Developmental Cognitive Neuroscience, Institute of Development, Aging and Cancer, Tohoku University, Sendai, Japan; 2 Division of Medical Neuroimaging Analysis, Department of Community Medical Supports, Tohoku Medical Megabank Organization, Tohoku University, Sendai, Japan; 3 Department of Radiology and Nuclear Medicine, Institute of Development, Aging and Cancer, Tohoku University, Sendai, Japan; 4 Department of Functional Brain Imaging, Institute of Development, Aging and Cancer, Tohoku University, Sendai, Japan; 5 Human and Social Response Research Division, International Research Institute of Disaster Science, Tohoku University, Sendai, Japan; 6 Smart Ageing International Research Center, Institute of Development, Aging and Cancer, Tohoku University, Sendai, Japan; 7 Graduate Schools for Law and Politics, The University of Tokyo, Bunkyo, Tokyo, Japan; 8 Japan Society for the Promotion of Science, Tokyo, Japan; 9 Faculty of Medicine, Tohoku University, Sendai, Japan

## Abstract

Sex-role egalitarianism (SRE) is the belief that the sex of an individual should not influence the perception of his or her rights, abilities, obligations, and opportunities. Thus, low SRE reflects a more conservative stereotypical view on sex-role. Here we investigated anatomical correlates of individual differences in SRE in the present study. We used voxel-based morphometry, a questionnaire to determine an individual’s SRE and associated psychological measures, and determined the association of SRE with gray matter structures and their cognitive nature in healthy individuals (375 men and 306 women; age, 20.6 ± 1.8 years). We demonstrated that higher SRE was associated with smaller regional gray matter density (rGMD) in the anterior part of the posterior cingulate cortex (PCC) and higher rGMD in the right amygdala. Post-hoc analyses revealed psychological measures characterized by contentious interpersonal orientations, such as contentious achievement motivation, were associated with lower SRE and higher rGMD in the anterior part of PCC. Depressive tendencies were associated with lower SRE and higher rGMD in the right amygdala. These findings suggest that variations in stereotype on sex role have roots in the limbic brain structures linked to contentious interpersonal orientation (cingulate) and negative mood (amygdala).

Sex-role egalitarianism (SRE) is the belief that the sex of an individual should not influence the perception of his or her rights, abilities, obligations, and opportunities^1^. More specifically, sex roles are behaviors, social expectations regarding personality, and the norms considered appropriate for men and women, and SRE is the belief of the equality of each sex toward sex roles^2^. Thus, low SRE reflects a more conservative stereotypical view on sex-role. SRE is quantified using self-reported questionnaires that consist of items measuring attitudes toward the marital, parental, employment, social-interpersonal, and educational roles of men and women^2^,^3^. SRE has become increasingly common^4^; however, substantial individual differences in SRE remain.

One method to quantify SRE is a short form of the Japanese version of the Scale of Egalitarian Sex Role Attitudes (SESRA-S)^2^, which is a self-report questionnaire that is used to measure an individual’s SRE. The SESRA-S contains 15 items assessing various attitudes that are related to SRE, such as attitudes toward (a) the association between men and women and role-sharing, (b) having, raising, and educating children, (c) women working, and (d) egalitarian values in society. One factor structure of this scale has been supported through factor analyses^2^.

On the other hand, a wide range of psychological studies have examined SRE using this scale and other scales. As predicted, from the definition, a previous study^5^ showed that lower SRE is associated with more frequent gender harassment (conduct that conveys hostile and degrading attitudes about women such as questioning a women’s competence in performing her job)^6^. Other studies revealed that lower SRE is associated with several types of sexual harassment^7^ and more frequent non-sexual violence against married or unmarried partners^8^,^9^. Lower SRE was also associated with tolerance toward rape and acceptance of myths regarding rape^10^,^11^ and with homophobia^12^. Furthermore, lower SRE appears to show a moderate-to-weak robust association with more negative emotional or personal characteristics. Such characteristics include a lower need for autonomy^13^, a higher need for social recognition^13^, and a wide range of mental health problems, such as alcoholism, depression, anxiety, and stress^8^,^14^. Stith and Farley^8^ investigated how marital violence of males was associated with other variables among the husband with problems related to violence. In that study, lower SRE was associated with higher levels of alcoholism and stress in marital life. Weatherill *et al*.^14^ investigated roles of women’s SRE in the adaptation of women to the military. They revealed that among women, higher SRE was weakly but robustly associated with lower depression, lower anxiety, and fewer post-traumatic stress disorder symptoms.

By definition, SRE is an individual characteristic that facilitates more egalitarian societies and on the other hand, lower SRE reflects a more conservative stereotypical view on sex-role. Furthermore, the SRE construct correlates uniquely with a wide range of behaviors and beliefs about men and women, making SRE an important research topic. However, despite the importance of SRE, the anatomical basis of SRE is unknown.

As described in our previous study^15^, both functional imaging studies and structural studies have advantages and disadvantages, but the findings from these two methods should complement one another. Structural imaging studies are especially useful for investigating the anatomical correlates of personal characteristics involving a wide range of behaviors or opinions that occur outside the laboratory, such as SRE. This is because unlike functional magnetic resonance imaging (fMRI) studies, the findings of structural imaging studies are not limited to the specific regions engaged in a task or that respond to stimuli during scanning. Furthermore, although fMRI studies do have advantages, they have the disadvantage that everyday tasks must be simplified so they can be performed within the constraints of an MRI scanner, which may lead to activation patterns in the brain that are different from those occurring outside the scanner^16^. Furthermore, in MRI correlation studies (including those of fMRI) that investigated the neural basis of individual differences, we are able to use established cognitive measures with proven reliability and validity to determine individual differences in cognition.

In general, lower SRE is associated with a wide range of psychological characteristics related to hostility and violence against other groups (social aggression), as well as negative emotional characteristics. Neuroimaging studies have revealed that several areas are associated with aggression, and among them, the amygdala is believed to play a central role in social aggression, possibly through excessive responses in this area (for review, see ref. 17). The gray matter of the amygdala is consistently reduced in disorders characterized by aggression^18^,^19^. Further, the gray matter in this area is associated with several negative emotional characteristics, such as depression^20^, stress^21^, anxiety^22^, as well as personality traits such as neuroticism^23^. The amygdala is involved in not only aggression and negative emotions but also social judgment^24^. Moreover, several studies have shown that the amygdala plays an important role in the generation of social bias, prejudice, and stereotypes^25^, (for review, see ref. 26). The structure of the amygdala also has been associated with political conservativeness^27^. These cognitions are apparently have some relevance to (low) SRE. Therefore, we hypothesized that individual differences in SRE would be associated with differences in the structure of the amygdala. However, several other areas have been linked to aggression or negative emotions, including the hippocampus^20^, insula^15^,^20^, anterior cingulate cortex^20^, posterior cingulate cortex (PCC)/precuneus^28^,^29^, and subgenual cingulate cortex^20^. Further, the described functional imaging studies suggested that the key role of the amygdala was in the generation of social bias, prejudice, and stereotypes. They also suggested the importance of (a) the areas of the network consisting of the dorsolateral, ventrolateral, frontal, and inferior parietal and the anterior cingulate cortices, perhaps in regulatory roles^25^,^26^, (b) areas of the default mode network for the expectation of social roles^30^, (c) areas of the orbitofrontal cortex for in group favoritism and outgroup negativity^25^. Most of these areas show anatomical and functional connections with the amygdala^31^. These wide range of structures are peripheral candidates of anatomical basis of SRE.

The purpose of this study is to investigate gray matter structural basis of SRE. Specifically, we used voxel-based morphometry (VBM) to investigate whether individual differences in SRE were associated with regional gray matter density (rGMD)^32^. We aimed to test our central hypothesis that the amygdala is involved in SRE through the region of interest hypothesis. However, the other potential candidates for the anatomical basis of SRE are diverse; therefore, we used a whole-brain analysis to investigate the anatomical basis of SRE in an exploratory manner.

## Results

### Basic data

Table 1 shows the mean and standard deviation (SD) for age and scores for RAPM and SESRA-S for men and women. Table 2 shows the distributions of SESRA-S scores for men and women.

A multiple regression analysis with the SESRA-S score selected as the dependent variable, and age, sex, and RAPM score selected as independent variables, revealed that women showed significantly higher SESRA-S scores than men. This finding is consistent with that of a previous study^2^. Note, in the multiple regression analysis with a dichotomous independent variable, the slope is the change in the dependent variable associated with a one-unit change in the independent variable. However, with only two categories, that value becomes the difference in the means between the first (female) and second (male) groups^33^.

We performed multiple regression analyses with the SESRA-S score selected as the dependent variable, and age, sex, and RAPM score selected as independent variables. One additional independent variable was selected from the following: trait anger (STAXI), anger-out (tendency to express anger toward others, STAXI), hostile behaviors (CTS), neuroticism (NEO-FFI), and suicide ideation and depressive tendencies (subscale of General Heath Questionnaire 30). These analyses revealed that the SESRA-S scores showed a significant negative correlation with trait anger, anger-out, hostile behaviors, neuroticism, and suicide ideation and depressive tendencies (Table 3).

Finally, we performed multiple regression analyses with one of the following selected as the dependent variable: trait anger (STAXI), anger-out (tendency to express anger toward others, STAXI), hostile behaviors (CTS), neuroticism (NEO-FFI), and suicide ideation and depressive tendencies (subscale of General Heath Questionnaire 30). Age, sex, and total intracranial volume (TIV) were selected as independent variables. These analyses revealed that TIV did not show significant correlation with any dependent variable (Table 4).

### Correlation between rGMD and SRE

**Figure 1:**
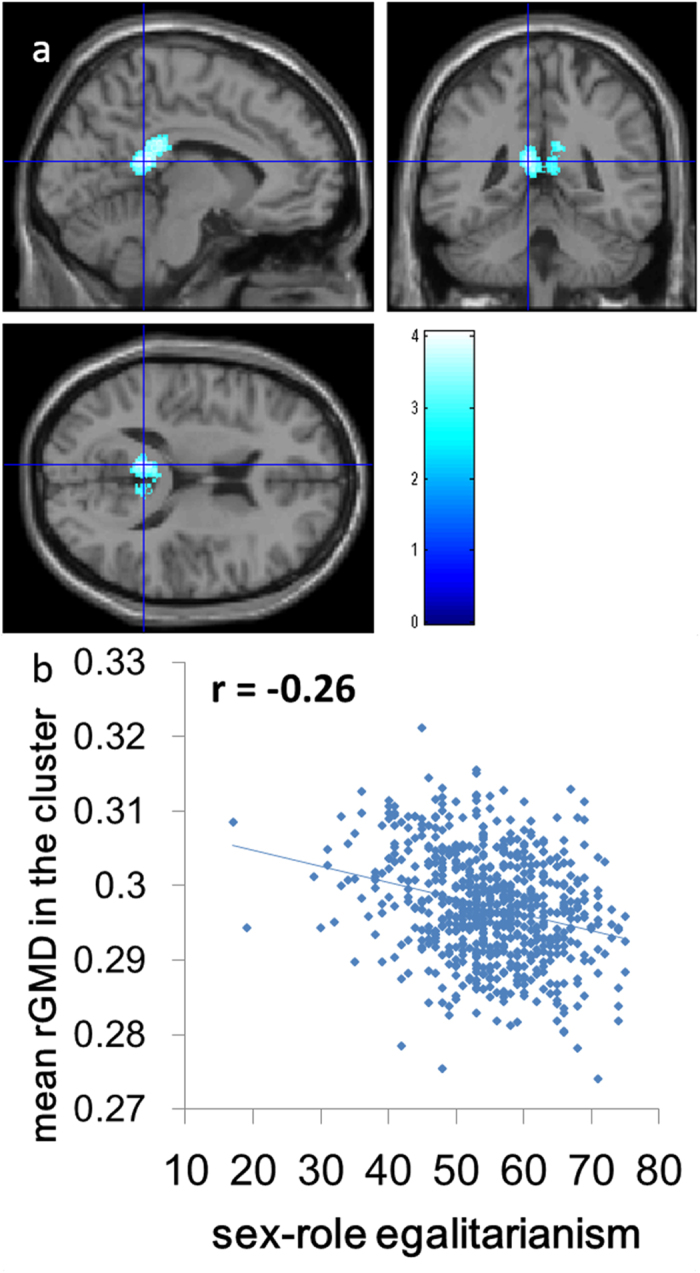
Anatomical correlates of SRE. (**a**) The region showing significant correlation is overlaid on a “single-subject” T1-weighted structural image of SPM5. The blue color represents the T score for the negative correlation between rGMD and the SESRA-S score. rGMD was negatively correlated with individual SRE in a cluster in the anterior part of the posterior cingulate cortex. Results are shown with *P* < 0.05, corrected for multiple comparisons at the non-isotropic adjusted cluster level with an underlying voxel level of *P* < 0.0025, uncorrected. (**b**) A scatter plot between the SESRA-S score and the mean rGMD value in the significant cluster in (**a**) has been shown for visualization only.

We investigated the association between rGMD and individual differences in SRE. A whole-brain multiple regression analysis including age, sex, RAPM score, and TIV revealed that the SESRA-S score was significantly and negatively correlated with rGMD in the anterior part of PCC (peak MNI coordinates *x, y, z* = −7.5, −43.5, 15; peak *t* value = 4.06; *P* < 0.001, corrected for multiple comparisons at the whole-brain level using the non-isotropic [non-stationary] adjusted cluster level with a cluster-determining uncorrected threshold of *P* < 0.0025; Fig. 1). There were no other significant results.

**Figure 2:**
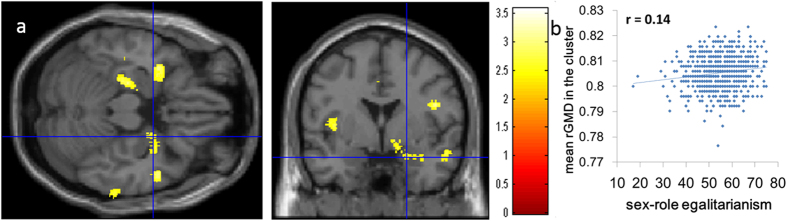
Anatomical correlates of SRE. (**a**) Regions showing a significant correlation are overlaid on a “single-subject” T1-weighted structural image of SPM5. The red color represents the T score for the positive correlation between rGMD and the SESRA-S score. rGMD was positively correlated with individual SRE in an area of the right amygdala. A similar tendency was seen in the corresponding area in the left hemisphere. Results are shown with *P* < 0.01, uncorrected, and are for visualization only. (**b**) A scatter plot between the SESRA-S score and the mean rGMD value in the significant cluster in (**a**) has been shown for visualization only.

Areas with a priori hypothesis with no significance at the whole-brain level were examined with ROI analyses and a small volume correction. After correcting for the effects of age, sex, RAPM score, and TIV, the SESRA-S score showed a significant positive correlation with rGMD in the right amygdala (peak MNI coordinates *x, y, z* = 22.5, 0, −21; peak *t* value = 2.77; *P* = 0.042, corrected for FWE at the voxel level within ROI, SVC for areas with a strong a priori hypothesis; Fig. 2). Similar, but non-significant correlation patterns were observed in the corresponding areas in the left hemisphere (Fig. 2). There were no other significant results.

### Interaction effects between SRE and sex on rGMD

ANCOVA performed using data for age, sex, RAPM score, and TIV from both sexes revealed no significant interaction effects of the SESRA-S score and sex on rGMD.

### Post-hoc analyses of the associations between rGMD of the identified significant clusters and psychological correlates of SRE

To reveal the nature of the associations between SRE and significant clusters, we extracted the mean rGMD from the significant clusters identified in each individual. Using these mean values, we investigated their associations with other variables. In these analyses, the dependent variables were mean rGMD values in these clusters and the independent variables were age, sex, RAPM score, TIV, and one of the psychological variables hypothesized to be related to SRE.

Multiple regression analyses revealed that the mean rGMD for the significant cluster identified in the anterior part of PCC showed tendencies toward a positive correlation with trait anger (*P* = 0.080, uncorrected; *P* = 0.087, corrected for FDR; *t* = 1.753), anger-out (*P* = 0.094, uncorrected; *P* = 0.087, corrected for FDR; *t* = 1.679), and hostile behaviors (*P* = 0.046, uncorrected; *P* = 0.054, corrected for FDR; *t* = 2.000), but not with neuroticism (*P* = 0.819, uncorrected; *P* = 0.500, corrected for FDR; *t* = 0.229) or suicide ideation and depressive tendencies (*P* = 0.489, uncorrected; *P* = 0.334, corrected for FDR; *t* = 0.692).

In our previous study, rGMD for the anterior part of PCC was positively correlated with competitive achievement motivation^15^. Competitive achievement motivation is the desire to handle and succeed in difficult tasks, and is directed at seeking social prestige by defeating and achieving better results than others, in addition to the psychological variables associated with contentiousness^15^. This previous study (N = 185) and the present study shared data from 89 subjects (Although the project is still ongoing and this study involves more subjects, we began gathering the data of the SESRA-S questionnaire after beginning gathering data on achievement motivation. Thus there is only partial overlap in subjects between two studies). Therefore, we investigated in a post-hoc manner the association between competitive achievement motivation and the mean rGMD for the significant cluster in the anterior part of PCC. A multiple regression analysis revealed, after correcting the effects of age, sex, RAPM score, and TIV, that the mean rGMD for this cluster showed a significant positive correlation with competitive achievement motivation (*P* = 0.006, uncorrected; P = 0.023, corrected for FDR; *t* = 2.737). After correcting the effects of age, sex, and RAPM score, the SESRA-S score showed a significant negative correlation with competitive achievement motivation (*P* = 1.555*10^−4^, uncorrected; *P* = 0.008, corrected for FDR; *t* = −3.804). Some part of PCC is associated with cognition related to social functions; therefore, we examined whether rGMD for this cluster was associated with sociality (agreeableness scale of NEO-FFI). A multiple regression analysis revealed, after correcting the effects of age, sex, RAPM score, and TIV, that the mean rGMD for this cluster did not correlate significantly with agreeableness (*P* = 0.485, uncorrected; *P* = 0.334, corrected for FDR; *t* = −0.699).

Finally, we performed multiple regression analyses with age, sex, RAPM score, TIV, and mean rGMD for the significant cluster identified in the right amygdala. Results showed that rGMD of this area had significant correlations with or a tendency toward a negative correlation with neuroticism (*P* = 0.093, uncorrected; P = 0.087, corrected for FDR; *t* = −1.683) and suicide ideation and depressive tendencies (*P* = 0.010, uncorrected; P = 0.016, corrected for FDR; *t* = −2.574), but not with trait anger (*P* = 0.812, uncorrected; P = 0.500, corrected for FDR; *t* = −0.239), anger-out (*P* = 0.187, uncorrected; *P* = 0.162, corrected for FDR; *t* = −1.322), hostile behaviors (*P* = 0.204, uncorrected; *P* = 0.166, corrected for FDR; *t* = −1.273), and competitive achievement motivation (*P* = 0.330, uncorrected; *P* = 0.252, corrected for FDR; *t* = 0.975). However, the association between neuroticism and rGMD for this cluster was female specific. A significant correlation with neuroticism was only observed when female data were analyzed (*P* = 0.023, uncorrected; P = 0.023, corrected for FDR; *t* = −2.281), and not when male data were analyzed (*P* = 0.938, uncorrected; *P* = 0.530, corrected for FDR; *t* = −0.077).

## Discussion

This study investigated the associations between brain structures and stereotype on sex role. We demonstrated that higher SRE, hence less stereotypical view on stereotype on sex role was associated with smaller rGMD in the anterior part of PCC. There were no interaction effects between SRE and sex for rGMD. Post-hoc analyses showed that higher rGMD for this area was associated with higher competitive achievement motivation and hostile behaviors of Type A personalities. In addition, we observed associations between higher competitive achievement motivation and hostile behaviors of Type A personalities and lower SRE. Thus, our results indicate that stronger stereotype on sex role is associated with the limbic structure of areas involved in contentious interpersonal orientation. Furthermore, consistent with our hypothesis, we demonstrated that higher SRE was associated with higher rGMD in the right amygdala. Post-hoc analyses revealed that higher rGMD in this area was associated with higher suicide ideation and depressive tendencies; lower SRE was associated with higher suicide ideation and depressive tendencies. Thus, our results suggest that stronger stereotype on sex role is associated with the limbic structure of the right amygdala, which may be related to this this region’s association with negative emotions. Most of the peripheral candidates of anatomical correlations of SRE did not show a significant correlation between rGMD and SRE. This may be because of a lack of statistical power as SRE is potentially associated with widespread areas, but this is not clear from the present study.

As described in the introduction, the amygdala has strong connections with a wide range of cortical and subcortical areas. In relation to the observed results, the amygdala and posterior cingulate cortex, together with supragenual cingulate cortex, are functionally and structurally connected and form a circuit^31^. The areas of this circuit are activated by emotional paradigms^34^ and affected structurally and functionally by affective disorders^20^. Thus, while the two identified areas have distinct functions, as described below, they also function as a circuit relevant to affection. Our results may be construed as critical roles of this affective circuit in SRE.

The present results suggest that stronger stereotype on sex role (lower SRE) is associated with higher rGMD in the anterior part of PCC, which is likely to be through this region’s association with contentious interpersonal orientation. Our psychological results revealed significant or tendencies of associations between lower SRE and contentious interpersonal orientation (competitive achievement motivation, hostile behaviors of Type A personalities), anger (trait anger), and social aggression (expression of anger toward others). These results are congruent with previous psychological studies that have repeatedly shown associations between lower SRE and violence or hostility toward others^8^,^9^,^10^,^11^,^12^. Further, not only lower SRE but also competitive achievement motivation and hostile behaviors of Type A personalities are associated with higher rGMD in the anterior part of PCC. Trait anger and expression of anger toward others also showed this tendency. The anterior part of PCC is functionally associated with emotion-related cognition, such as anger, fear, and pain^28^. Our previous study also showed that rGMD in the anterior part of PCC was associated with competitive achievement motivation, which used a subject sample that was largely different from that used in the present study (only 89 subjects overlapped)^15^. However, it is difficult to determine whether higher or smaller rGMD in the medial prefrontal and parietal regions is associated with better functioning in these areas in young adults (for full discussions related to this and summary of relevant previous studies, see ref. 15). Regardless, these findings point to an association between contentious interpersonal orientation and higher rGMD in the anterior part of PCC. A lack of serotonin is believed to play a key role in aggression for review^17^, and reduced serotonin in the primate PCC has been associated with greater social aggression and unfriendliness^29^. Although lower SRE was also associated with depressive tendencies and neuroticism, these cognitive characteristics were not associated with rGMD of the anterior part of PCC. Although areas in PCC and the precuneus play important roles in socio-related cognitive functions^35^, rGMD in the anterior part of PCC did not associate with traits related to sociality (agreeableness) in the present study. The present and previous findings point to the interpretation that lower SRE, hence stronger stereotype on sex role is associated with higher rGMD in the anterior part of PCC through this region’s association with contentious interpersonal orientation.

The present results for the anterior part of PCC may provide new insights into the nature of stronger stereotype on sex role. The anterior part of PCC seems to be best characterized with contentious interpersonal orientation, and these psychological characteristics seem to be associated with lower SRE score. Thus, lower SRE may have a nature of contention or rivalry over territory between the two sexes. Men who have lower SRE may have this idea because they have to win the competition in the workplace, and to do so, they must expel women from the workplace. The same idea may hold true for women, and women who have lower SRE may think that their existence is threatened by man’s participation in jobs traditionally performed by women.

The present findings suggest that stronger stereotype on sex role (lower SRE) is associated with lower rGMD in the right amygdala through this region’s association with depressive tendencies. Our psychological findings revealed associations between lower SRE and negative emotional characteristics, such as neuroticism or suicide ideation and depressive tendencies. These findings are congruent with previous psychological studies^8^,^14^. Not only lower SRE but also suicide ideation and depressive tendencies are associated with lower rGMD in this part of the right amygdala; neuroticism showed the same tendency. The amygdala participates in a wide range of functions, including positive and negative emotions and sexual behaviors^36^. However, stimulation of the amygdala can evoke anxiety and fear, whereas removal of the amygdala leads to a loss of such emotions^36^. Associations between reduced gray matter in this area and a wide range of negative emotional characteristics have been shown repeatedly in clinical and non-clinical studies. These negative emotional characteristics include depression^20^, stress^21^, and anxiety^22^, in addition to negative personality traits, such as those related to neuroticism^23^. Although, lower SRE was also associated with aggression and contentious cognitive components, these cognitive characteristics were not associated with rGMD in this part of the right amygdala. Previously, individuals with a psychological disorder characterized with aggressiveness^37^ has been repeatedly shown to have less rGMD in the amygdala^18^,^19^, and this structural characteristic has been linked to the display of their aggressiveness^17^. In the present study, however, aggressiveness was not strongly or significantly associated with rGMD in this amygdala area. On the other hand, borderline personality disorder has also been characterized by negative emotional characteristics, such as anxiety and depression^37^. Future studies should be conducted to determine whether rGMD in the right amygdala is associated with depressive tendencies rather than aggression in individuals with borderline personality disorder.

The present results regarding the right amygdala may provide new insight and speculations on how to alleviate stereotype on sex role. The important aspect is to improve mental health problems through a wide range of interventional methods such as aerobic exercise and cognitive training, which are known to improve these problems^38^,^39^. The present study was a cross-sectional study; thus, there are limitations in indicating causality. However, the amygdala is associated with negative emotions and improvements in negative emotions is associated with an increase in the density of the right amygdala^21^. Therefore, improving negative mood may prevent stereotype on sex role and may mitigate a wide range of problems associated with lower SRE and stereotypical view on sex role.

As for the psychological results, the present findings generalized the previous findings of associations of SRE with contentious interpersonal orientations related to sex and gender rather than those unrelated to sex or gender. The present findings also generalized the association between low SRE and the negative emotional characteristics in certain groups to general populations. Previous studies have shown an association between lower SRE and a wide range of contentious opinions or behaviors related to harassment and violence related to gender or sex^6^,^7^,^8^,^9^,^10^,^11^,^12^. Previous studies have also investigated the association between lower SRE and a wide range of negative emotional characteristics. However, other than the study by King and King^13^, those investigations were confined mainly to certain groups, such as husbands involved in marital violence and associated problems^8^ or women during adaptation to military^14^. These previous findings did not show a specificity for SRE’s association with these areas related to sex or gender. Nonetheless, whether the association of SRE with contentious interpersonal orientation and negative emotional characteristics can be generalized beyond these areas or subjects remains unclear. In this study, we showed that the associations between SRE and these characteristics are not limited to the areas or subjects in normal samples. The correlations were not high, and other factors such as education^2^, family, or parental environmental factors may have had a impact on an individual’s SRE. Nonetheless, these unspecific individual psychological characteristics do associate robustly with SRE. The present neuroimaging findings showed that SRE has roots in neural characteristics unrelated to specific cognitive tasks related to gender or sex (such as brain structures, and not task-related functional activation). As with those previous neural findings, the present findings indicate that SRE has roots in an corresponding individual’s cognitive characteristics that are not specifically related to sex or gender, even in normal populations.

One may say that SRE is likely to be affected by cultural and environmental factors and inherited to a lesser extent. And Further, regional gray matter volume is highly heritable. Thus, the association between the two or their investigation may not have any substantial meaning. However, this idea needs to be viewed with caution. First, consider the notion that SRE is likely to be affected by cultural and environmental factors and may be less heritable. Although this is a completely plausible opinion, the same can be said of political orientation (too conservatism). However, a twin study showed that for the overall index of political conservatism, genetics accounted for approximately half of the variance while a shared environment, including parental influence, accounted for only 11%^40^. So, how much of one trait is actually inherited is quite unpredictable from a common sense point of view, and the abovementioned finding has astonished the relevant field^41^. Unfortunately, we are not aware of any studies that report the heritability of SRE. On the other hand, experts in twin studies suggest, based on the overall views of the findings in the areas that most individual traits or abilities show a substantial contribution from genetic and non-shared environments and a relatively lower contribution from shared environments^42^. This means that we cannot presume a low genetic contribution to individual traits. One may also say that since SRE is quite likely related to mate choice and procreation strategy, it is essentially biological in nature and thus may also have a root in the brain structure. As for traits that can be theoretically related to SRE, even in preschool children who are generally susceptible to environmental effects e.g.,^43^, gender role behaviors have a genetic contribution of an average of 45% in both sexes^44^. This is despite the findings that parents’ ideas and behaviors regarding gender role egalitarianism affect children’s sex-typed behaviors^45^. Given that genetic twin studies have generated findings that were beyond our traditional common sense, we believe we cannot presume that the genetic contribution to individual differences of SRE is trivial, and as such, it is an open scientific question. The second point is the notion that the brain regional volume is known to have a significant genetic contribution. It is true that an earlier study using a small sample and voxel-based data showed astonishing heritability in regional gray matter volume^46^. However, voxel-based analyses combined with small samples should never be used to estimate the regional strength of the effect^47^. On the other hand, a more recent study using a large sample dataset of regions of interest showed the heritability of the gray matter volume was mostly within the range of 30%–70%^48^ and the area in the medial temporal lobe, which is known to be affected by many environmental factors, learning, and diseases^20^,^49^, showed a heritability of 30%^48^. The third point is the notion that one measure is highly heritable so that any investigation into the association of that measure with factors that can be strongly related to environmental factors may not mean much. However, the heritability of body mass index was estimated to be around 70%^50^, yet an investigation on the factors affecting or changing the body mass index is quite an important topic in health science. Therefore, we do not believe a heritability of about 70% in individual differences negates the meaning of such investigations.

This study had a few limitations. One limitation that is common to our previous studies and other studies using college cohorts^15^,^51^ is the limited sampling from young, healthy subjects with a high educational background. Limited sampling from the full range of intellectual abilities is a common hazard when sampling from college cohorts^51^. Limited sampling may be an important step to rule out the effects of age or education level that could strongly influence the brain structures and increase the sensitivity of the analyses. Nevertheless, SRE is associated with the education level as well as age. Whether our findings would remain true in a full-range population sample must be determined with larger and more representative samples. In addition, because of the fundamental nature of the whole-brain analysis, we could only report strong correlations between anatomical structures and SRE that surpassed a stringent threshold or that had a strong a priori hypothesis. However, there may be weakly distributed anatomical correlates of SRE throughout the brain that we could not identify. Finally, we did not perform official, prolonged diagnostic procedures for all existing diseases in our exclusion criteria. Thus, it is possible that some subjects could have had an undiagnosed disease. However, the most prevalent psychiatric conditions seem to be mood disorders, anxiety disorders, social phobia, and post-traumatic stress disorder and patients with such conditions may choose not to participate in MRI experiments. Nonetheless, this type of problem may be common among imaging studies using non-clinical subject samples.

SRE is an essential individual characteristic that facilitates egalitarian societies and on the other hand, lower SRE reflects more conservative stereotypical view on sex-role. Furthermore, the SRE construct is uniquely correlated to a wide range of behaviors affecting the welfare of men and women. Understanding the neural basis of SRE and stereotype on sex role could provide new insight into the nature of stereotype on sex role and how to alleviate stereotype on sex role to achieve egalitarian societies.

## Methods

### Subjects

Six hundred and eighty-one healthy, right-handed individuals (375 men and 306 women; 20.6 ± 1.8 years) participated in this study as part of an ongoing project investigating associations among brain imaging, cognitive functions, aging, genetics, and daily habits e.g.^15^,^52^. For details of subjects’ characteristics, inclusion and exclusion criteria, recruitment, see Supplemental Methods. Written informed consent was obtained from each subject in accordance with the Declaration of Helsinki (1991). This study was approved by the Ethics Committee of Tohoku University.

### The Scale of Egalitarian Sex Role Attitudes-Short Form (SESRA-S)

The SESRA-S contains 15 items that are answered by subjects selecting responses ranging from “I don’t agree at all,” to “I agree very much.” The SESRA-S is scored with a 5-point Likert scale and probes for the following tendencies related to SRE:

1. attitudes toward the association between men and women and role sharing (e.g., household labor should be a collaborative work between sexes);
2. attitudes toward having, raising, and educating children (e.g., raising children is the most important career for women; reverse item);
3. attitudes toward women working (e.g., women need not work unless they are economically inconvenienced; reverse item); and
4. attitudes toward egalitarian values in society (e.g., we should stress the right and duty that ensure equality between the sexes at home and in society);

In the present analysis, the answers were compiled into a single score (responses from reverse items were added after being calculated as 6−x), with higher scores indicating higher SRE.

The reliability and validity of the scale has been well previously shown. The correlation coefficient between the full-scale SESRA and SESRA-S scores was *r* = 0.94^2^. The internal consistency of the SESRA-S, as measured with Cronbach’s coefficient α, was 0.91, and the test–retest reliability of SESRA-S was 0.89^2^. These results indicate a high reliability of SESRA-S. Consistent with the previous findings of SRE, women typically show a higher SESRA-S score than men^2^, and a higher education level is associated with a higher SESRA-S score^2^. Further, women with jobs show higher SESRA-S scores than those without jobs^2^. Among the wide range of age, higher age is associated with a lower score of SESRA-S, and support for changing one’s name after marriage is also associated with a higher SESRA-S score^2^. These results support the criterion-related validity of SESRA-S.

The number of items used for assessing SRE may seem too small to produce reliable data. However, the reliability of the questionnaire can be estimated by, for example, Cronbach’s coefficient α, which is determined by both the length of the questionnaire and strength of the correlations among the items or factors it includes. In our current questionnaire, the Cronbach’s coefficient α was 0.91^2^. A Cronbach’s coefficient of α > 0.65 is considered sufficiently reliable^53^, meaning that our data using this scale is highly reliable.

### Other psychological measures

Raven’s Advanced Progressive Matrix RAPM^54^; was used to assess intelligence^54^ and adjust for the effect of general intelligence on brain structures. For details of the administration of this test, see Supplemental Methods.

We also used several questionnaires to assess the individual characteristics hypothesized to be associated with SRE. To assess hostile behaviors, we used a hostile behaviors subscale of the *Coronary-Prone Type Scale* for Japanese CTS^55^, which is a Type A behavior pattern scale for Japanese. To assess the trait anger and aggression toward others, we used the anger and anger-out subscales of the *State-Trait Anger Expression Inventory* (STAXI)^56^, which determines how often anger is expressed toward other people or objects. Negative emotion-related personality characteristics, namely neuroticism, was assessed using the *NEO Five-Factor Inventory* (NEO-FFI)^57^. NEO-FFI is a self-administered measure of normal personality functioning. We assessed negative emotions using the depression and suicide ideation subscale of *General Heath Questionnaire 30*^58^. However, we did not include these negative emotion measures in the whole-brain multiple regression analyses investigating the association between SRE and rGMD. For the rationales of this method, see Supplemental Methods.

### Image acquisition and analysis

All MRI data acquisition was performed using a 3-T Philips Achieva scanner. High-resolution T1-weighted structural images (T1WIs: 240 × 240 matrix, TR = 6.5 ms, TE = 3 ms, FOV = 24 cm, slices = 162, slice thickness = 1.0 mm) were collected using a magnetization-prepared rapid gradient echo sequence.

### Preprocessing of T1-weighted structural data

The strength of VBM was presented in Supplemental Methods.

Preprocessing of the structural data was performed using Statistical Parametric Mapping software (SPM8; Wellcome Department of Cognitive Neurology, London, UK) implemented in Matlab (Mathworks Inc., Natick, MA, USA). Using modified new segmentation algorithm and Diffeomorphic Anatomical Registration Through Exponentiated Lie Algebra (DARTEL) registration process, T1-weighted structural images obtained for each subject were segmented, normalized (without modulation) to the Montreal Neurological Institute (MNI) space to images with 1.5 × 1.5 × 1.5 mm^3^ voxels. For details of these procedures, see Supplemental Methods. In accordance with the developers of VBM softwares, we called these images GMD images (http://dbm.neuro.uni-jena.de/vbm/segmentation/modulation/). Subsequently, all images were smoothed by convolving them with an isotropic Gaussian kernel of 12 mm full-width at half-maximum (FWHM). This larger smoothing value was required in relation with the non-isotropic adjusted cluster size test^59^ that was required in VBM analyses. For details, see Supplemental Methods.

### Statistical analyses

We investigated whether rGMD was associated with individual differences in SRE. Statistical analyses of morphological data were performed using SPM5 and VBM5 software (http://dbm.neuro.uni-jena.de/vbm/), an extension of SPM5 for the reasons described below. In these analyses, we included only voxels that showed rGMD values > 0.10 in all subjects. The primary purpose for using gray matter thresholds was to cut the periphery of gray matter areas and to effectively limit the areas analyzed to those most likely to be gray matter. The voxels outside the brain areas are more likely to be affected by signals outside the brain through smoothing. Masking the analysis to the brain areas is performed in fMRI analyses of SPM by default.

In the whole-brain multiple regression analyses, we tested for a relationship between SRE, as assessed by SESRA-S, and rGMD. The analyses were performed with sex, age, RAPM score, and TIV (total gray matter volume + total white matter volume + total cerebral spinal fluid volume and calculated by the sum of the total signals of normalized images of regional gray matter volume, regional white matter volume, and regional cerebrospinal fluid volume in the intracranial areas), which is arguably the most widely used global measure for global brain effects as additional covariates, resulting in 5 covariates in total. When the total brain volume is included as a covariate in the analyses of density measures, these analyses examine the density of tissues that cannot be explained by the total brain volume. Age is included as a covariate despite the lack of significant association between SRE and age among this narrow range of age range (see Table 1). This is because age and brain structures are very strongly associated^52^ and in the model to explain the brain structures, including age should strongly increase the precision of the model. So, age almost always had better be included as covariates for cross-sectional brain structural analyses, regardless of age is associated with psychological variables of interest or not.

We investigated whether the relationship between rGMD and the SESRA-S score differed between sexes (whether the interaction between sex and the SESRA-S score affected rGMD). In the whole-brain analysis, we used a voxel-wise analysis of covariance (ANCOVA) in which sex was a group factor (using the full factorial option of SPM5). In this analysis, age, RAPM score, SESRA-S score, and TIV were covariates. These covariates, except for TIV, were modeled so that each covariate’s relationship with rGMD could be identified for each sex (using the interactions option in SPM5), which would allow investigation of the interaction effects of sex and the other covariates. TIV was modeled so that it had a common relationship with rGMD between sexes. The interaction between sex and SESRA-S score on rGMD (contrasts of [the effect of SESRA-S score for males, that for females] were [−0.5 0.5] or [0.5 −0.5]) were assessed using t-contrasts. These analyses of interaction effects between the SESRA-S score and sex are irrelevant to the purpose of this study and are performed for the interest of the readers. For details, see Supplemental Methods.

The statistical significance level was set at *P* *<* 0.05, corrected at the non-isotropic adjusted cluster level^59^ with an underlying voxel level of *P* *<* 0.0025. The rationale for this statistical method was provided in the Supplemental Methods.

Furthermore, for areas with a strong a priori hypothesis, namely the amygdala, the statistical significance level was set at *P* *<* 0.05, with small volume correction for multiple comparisons (family-wise error, FWE) in regions of interests (ROIs). The reasons for choice of ROIs were described in detail in the Introduction. All ROIs were constructed using the WFU PickAtlas Tool (http://www.fmri.wfubmc.edu/cms/software#PickAtlas) and were based on the Brodmann option of the PickAtlas. The masks of the amygdala were constructed bilaterally using this option. The Pickatlas ROI, which is not based on the single-subject brain structure, is on the MNI space and since the normalized images in this study are also on MNI space, the use of the Pickatlas ROI is not problematic.

The behavioral data were analyzed using the Predictive Analysis SoftWare release version 18.0.0 (PASW Statistics 18; SPSS Inc., 2010). Post-hoc analyses using the mean rGMD value within the significant clusters identified through the abovementioned analyses and associated psychological variables were also performed using this software. Eight analyses investigated the association between SRE and other psychological variables in this study (Table 3). Six of the analyses investigated the association between TIV and psychological variables in this study (except age and sex; Table 4). The analyses detailed on Table 4 are rather irrelevant to the study theme and are provided to show TIV itself are not mediating the associations of the study theme. There are also 16 analyses in the *post-hoc analyses of the associations between rGMD of the identified significant clusters and psychological correlates of SRE* results subsection for relevant analyses. In all these analyses using PASW, except the abovementioned eight analyses involving TIV that are irrelevant to the study theme, results with a threshold of *P* < 0.05, which were corrected for false discovery rate (FDR) using the graphically sharpened method^60^, were considered statistically significant. The correction for multiple comparisons using this method were applied to the results of the abovementioned 24 (8 + 16) multiple regression analyses.

## Additional Information

**How to cite this article**: Takeuchi, H. *et al*. Amygdala and cingulate structure is associated with streotype on sex-role. *Sci. Rep*. **5**, 14220; doi: 10.1038/srep14220 (2015).

## Supplementary Material

Supplementary Information

## Figures and Tables

**Table 1:** Demographic variables of study participants.

Measure	Males			Females		
	Mean	SD	Range	Mean	SD	Range
Age	20.74	1.97	18–27	20.45	1.63	18–27
RAPM	28.92	3.82	15–36	28.25	3.70	15–36
SESRA-S	52.10	8.36	17–72	58.67	7.81	35–75

**Table 2:** Distribution of SESRA-S scores among the study participants.

	–19	20–29	30–39	40–49	50–59	60–69	70–
SESRA-S (male)	2	1	21	106	177	64	4
SESRA-S (female)	0	0	4	28	139	111	24

**Table 3:** Statistical values for multiple regression analyses between the SRE score and other psychological variables.

Variables	Covariates inthe multipleregression analyses	*P* value (uncorrected,corrected for FDR)	*t* value	Standardizedpartial regressioncoefficient (β)
Age	Sex, RAPM	0.846, 0.500	−0.195	−0.07
Sex (Female = 1, Male = 0)	age, RAPM	1.31*10^−23^, 1.70*10^−22^	10.04	0.373
Raven’s Advanced Progressive Matrix (RAPM)	age, sex	0.993, 0.538	0.008	2.94*10^−4^
Trait anger (STAXI)	age, sex, RAPM	0.001, 0.003	−3.215	−0.115
Anger-out (STAXI)	age, sex, RAPM	0.007, 0.013	−2.698	−0.097
Hostile behaviors (CTS)	age, sex, RAPM	0.001, 0.003	−3.446	−0.123
Neuroticism	age, sex, RAPM	0.005, 0.013	−2.789	−0.101
Suicide ideation and depressive tendency	age, sex, RAPM	0.023, 0.030	−2.281	−0.081

Each statistical value represents the association between the SRE score and individual variables that are desribed in the row “Variables” in the multiple regression analyses. In each mutiple regression analysis, the dependent variable is the SRE score and the independent variables are one variable from the row “Variables” and individual variables that are described in the corresponding row of “Covariates in the multiple regression analyses”.

**Table 4:** Statistical values for multiple regression analyses between TIV and psychological variables.

Variables	Covariates in themultiple regressionanalyses	*P* value	*t* value	Standardized partialregression coefficient(β)
SRE	age, sex	0.573	0.564	−0.026
Trait anger (STAXI)	age, sex	0.242	1.172	0.058
Anger-out (STAXI)	age, sex	0.878	0.153	0.008
Hostile behaviors (CTS)	age, sex	0.490	0.691	0.034
Neuroticism	age, sex	0.651	0.452	0.022
Suicide ideation and depressive tendency	age, sex	0.845	−0.196	−0.010

Each statistical value is the association between TIV and the individual variables that are described in the row “Variables” in the multiple regression analyses. In each mutiple regression analysis, the dependent variable is TIV and the independent variables are one variable from the row “Variables” and individual variables that are described in the corresponding row of “Covariates in the multiple regression analyses”.
