# Supplementary material for: Amygdala and cingulate structure is associated with stereotype on sex-role
Source: Sci Rep. 2015 Sep 30;5:14220. doi: 10.1038/srep14220 (PMC4588577; doi:10.1038/srep14220)
Supplement: Supplementary Information [file srep14220-s1.pdf]

Supplemental Information

Amygdala and cingulate structure is associated with stereotype on sex-role

Hikaru Takeuchi*[a], Yasuyuki Taki[a,b,c], Atsushi Sekiguchi[b,d], Rui Nouchi[e], Yuka Kotozaki[f], Seishu Nakagawa[d], Carlos Makoto Miyauchi[d,g], Kunio Iizuka[d], Ryoichi Yokoyama[d,h], Takamitsu Shinada[d], Yuki Yamamoto[d], Sugiko Hanawa[d], Tsuyoshi Araki[f], Hiroshi Hashizume[a], Keiko Kunitoki[i], Yuko Sassa[a], Ryuta Kawashima[a,d,f]

[a]*Division of Developmental Cognitive Neuroscience, Institute of Development, Aging and Cancer, Tohoku University, Sendai, Japan*

[b]*Division of Medical Neuroimaging Analysis, Department of Community Medical Supports, Tohoku Medical Megabank Organization, Tohoku University, Sendai, Japan*

[c]*Department of Radiology and Nuclear Medicine, Institute of Development, Aging and Cancer, Tohoku University, Sendai, Japan*

[d]*Department of Functional Brain Imaging, Institute of Development, Aging and Cancer, Tohoku University, Sendai, Japan*

[e]*Human and Social Response Research Division, International Research Institute of Disaster Science, Tohoku University, Sendai, Japan*

[f]*Smart Ageing International Research Center, Institute of Development, Aging and Cancer, Tohoku University, Sendai, Japan*

[g]*Graduate Schools for Law and Politics, The University of Tokyo, Bunkyo, Tokyo, Japan*

[h]*Japan Society for the Promotion of Science, Tokyo, Japan*

[i] *Faculty of Medicine, Tohoku University, Sendai, Japan*

***Corresponding author:**

Hikaru Takeuchi

Division of Developmental Cognitive Neuroscience, IDAC, Tohoku University

4-1 Seiryo-cho, Aoba-ku, Sendai 980-8575, Japan

Tel/Fax: +81-22-717-7988

E-mail: takehi@idac.tohoku.ac.jp

Supplemental Methods

Details of subjects' characteristics, inclusion and exclusion criteria, recruitment

Data derived from the subjects in this study will be used in other studies irrelevant to the theme of this study; some subjects who participated in this study also participated in intervention studies (psychological and imaging data used in this study were recorded before the intervention). Psychological tests and MRI scans not described in this study were performed together with those described in this study. All subjects were university, college, or postgraduate students, or recent graduates (<1 year) from these institutions and had normal vision. The subjects were recruited with advertisements introducing the study that were posted on bulletin boards at Tohoku University or sent to potential subjects via email. These advertisements and emails also specified the study's exclusion and inclusion criteria. These exclusion criteria included handedness (left-handedness),

the existence of metal in and around the body, claustrophobia, use of certain drugs (antipsychotic drugs, illicit psychoactive drugs, other drugs for psychiatric and neurological that are prescribed by doctors and so on), a history of certain psychiatric or neurological disease, and previous participation in related experiments. We provided self-report questionnaires to each potential subject to assess their history of psychiatric illness and recent drug use. The questionnaires required subjects to provide a detailed list of any recent drug use. No subject had neurological or psychiatric illnesses (due to the exclusion criteria). The assessments performed during and after recruitment were voluntary self-report. Handedness was evaluated using the *Edinburgh Handedness Inventory* [1]. The score of > 0 toward right handedness was used as cut-off value, as has been done previously [2]. Written informed consent was obtained from each subject in accordance with the Declaration of Helsinki (1991). This study was approved by the Ethics Committee of Tohoku University.

Assessment of psychometric measures of general intelligence

Raven's Advanced Progressive Matrix RAPM; [3] was used to assess intelligence [3] and adjust for the effect of general intelligence on brain structures. As described similarly in our previous studies [4,5], Raven's Advanced Progressive Matrix [3] contains 36 nonverbal items requiring fluid reasoning ability. Each item consists of a 3 × 3 matrix with a

missing piece to be completed by selecting the best of 8 alternatives. The score of this test (number of correct answers in 30 min) was used as an index of individual psychometric measure of intelligence. The RAPM was administered in a group setting in this study. The RAPM tests can be administered individually by a psychologist or trained test administrator, or administered on a group basis [3].

The rationales for the model of the whole brain analyses

We did not include negative emotion measures in the whole-brain multiple regression analyses investigating the association between SRE and rGMD. This is because we did not regard these measures as "confounding variables." This is common in brain imaging correlation analyses of working memory capacity that do not include psychometric intelligence as a covariate e.g., [6] and brain imaging analyses of schizophrenia that do not include working memory capacity as a covariate e.g., [7]. This is also common in whole-brain analyses of depression that do not include neuroticism as a covariate [e.g., 8]. Instead, we regard these measures and SRE as having common or partially overlapping neural and cognitive bases that should not be and cannot be regressed out. For the same reasons, we believe that mediation analyses do not best fit our model or assumptions.

Strength of VBM

As summarized in our previous study [9], potential correlates of gray matter signals in VBM may include the number and size of neurons and glial cells, the level of synaptic bulk, and the number of neurites, [10, 11]. However, this notion remains to be proven by histological studies. Gray matter structures are associated with various cognitive abilities, and investigation of these associations can identify the brain regions associated with specific cognitive abilities or characteristics e.g., [5, 12]. Structural imaging thus provides unique and distinctive information about the neural origin of individual cognitive characteristics.

Segmentation and normalization processes of VBM

Using our new segmentation algorithm implemented in SPM8, T1-weighted structural images obtained for each subject were segmented into 6 tissues. In this process, the gray matter tissue probability map (TPM) was manipulated from maps implemented in the software so that the signal intensities of voxels (gray matter tissue probability of the default tissue gray matter TPM + white matter tissue probability of the default TPM) < 0.25 became 0. When this manipulated gray matter TPM is used, the dura matter is less likely to be classified as gray matter (compared with when the default

gray matter TPM is used), without other substantial segmentation problems. The default parameters were used in this new segmentation process, except that affine regularization was performed using the International Consortium for Brain Mapping template for East Asian brains. We then proceeded to the Diffeomorphic Anatomical Registration Through Exponentiated Lie Algebra (DARTEL) registration process implemented in SPM8. In this process, we used DARTEL imported images of the 5 TPMs (we didn't use the sixth image which mainly consisted of air space outside the brain) obtained with our abovementioned new segmentation process. First, the template for the DARTEL procedures was created using imaging data from 63 subjects who participated in a previous experiment in our laboratory and who participated in this project [13] and who have the same characteristics as the subjects in this study. As described previously [14], the first reason why we created the DARTEL template from images of the subjects and not from images of all subjects is because N = 63 is not a small number to create template compared with much of the previous studies and thus cannot be considered to be problematic. The second reason is the project which was introduced in subjects subsection and in which subjects of this study participated, is still ongoing and, DARTEL processes take huge amount of time and resultant images require storage resources, and everytime we change the number of subjects we cannot reprocess images

of all subjects and add newer images based on the different number of subjects to our storage.

Using this template, the DARTEL procedures were performed for all subjects in the present study using the default parameter settings. The resulting images were spatially normalized to the Montreal Neurological Institute (MNI) space to images with $1.5 \times 1.5 \times 1.5$ mm^3 voxels. We did not perform a volume change correction (modulation) by modulating each voxel with the Jacobian determinants derived from spatial normalization [15].

The reasons why we did not assume sex differences of anatomical correlates of SRE

These analyses of interaction effects between the SESRA-S score and sex are irrelevant to the purpose of this study and are performed for the interest of the readers. The study background does not necessarily make us formulate hypotheses of the interaction effects of sex and SRE on rGMD and our previous studies mostly failed to find interaction effects between sex and cognitive differences of neural systems regardless that cognitions show sex differences [16-22]. Further, sex differences of neural bases of cognition are popular but elusive concepts [23]. Thus, the results were treated in an exploratory manner and multiple comparison corrections were not applied.

Rationales for our statistical threshold and smoothing value of the whole brain imaging analysis

In this non-isotropic cluster-size test of the random field theory, a relatively higher cluster-determining threshold combined with high smoothing values of 12 mm was shown to lead to appropriate conservativeness in real data [24]. With high smoothing values, an uncorrected voxel-level threshold of $P < 0.01$ seems to lead to less conservative cluster level statistical values, whereas that of $P < 0.001$ seems to lead to slightly conservativeness cluster level statistical values [24]. This cluster-determining threshold ($P < 0.0025$) has been used in a number of studies of VBM [16, 25-33]. We used the VBM5/SPM5 software version for this test. This is because a previous validation study of this test using a real dataset [24] showed that the conditions of this non-isotropic adjusted cluster-size test were limited and depended on the smoothness of the data, as described above. However, there are substantial differences in the way that SPM8 and SPM5 estimate the actual FWHM in the areas analyzed, and this directly affects the cluster test threshold. Therefore, regardless of whether SPM5 or SPM8 is appropriate, our view is that the conditions for this non-isotropic adjusted cluster-size test described in the previous study [24] are no longer guaranteed in SPM8. This is because they are

different analyses and produce substantially different results.

References

1. Oldfield RC. The assessment and analysis of handedness: the Edinburgh inventory. *Neuropsychologia* 1971; **9**(1)**:** 97-113.

2. Westerhausen R, Kreuder F, Santos Sequeira SD, Walter C, Woerner W, Wittling RA *et al.* The association of macro-and microstructure of the corpus callosum and language lateralisation. *Brain Lang* 2006; **97**(1)**:** 80-90.

3. Raven J. *Manual for Raven's progressive matrices and vocabulary scales*. Oxford Psychologists Press: Oxford, 1998.

4. Takeuchi H, Taki Y, Sassa Y, Hashizume H, Sekiguchi A, Fukushima A *et al.* White matter structures associated with creativity: Evidence from diffusion tensor imaging. *Neuroimage* 2010; **51**(1)**:** 11-18.

5. Takeuchi H, Taki Y, Sassa Y, Hashizume H, Sekiguchi A, Fukushima A *et al.* Regional gray matter volume of dopaminergic system associate with creativity: Evidence from voxel-based morphometry *Neuroimage* 2010; **51**(2)**:** 578-585.

6. Hampson M, Driesen NR, Skudlarski P, Gore JC, Constable RT. Brain connectivity related to working memory performance. *J Neurosci* 2006; **26**(51)**:** 13338.

7. Honea R, Crow TJ, Passingham D, Mackay CE. Regional deficits in brain volume in schizophrenia: a meta-analysis of voxel-based morphometry studies. *AJ Psychiatry* 2005; **162**(12)**:** 2233-2245.

8. Peng J, Liu J, Nie B, Li Y, Shan B, Wang G *et al.* Cerebral and cerebellar gray matter reduction in first-episode patients with major depressive disorder: a voxel-based morphometry study. *Eur J Radiol* 2011; **80**(2)**:** 395-399.

9. Takeuchi H, Taki Y, Sassa Y, Hashizume H, Sekiguchi A, Nagase T *et al.* Regional gray and white matter volume associated with Stroop interference: Evidence from voxel-based morphometry. *Neuroimage* 2012; **59**(3)**:** 2899-2907.

10. May A, Gaser C. Magnetic resonance-based morphometry: a window into structural plasticity of the brain. *Curr Opin Neurol* 2006; **19**(4)**:** 407-411.

11. Takeuchi H, Taki Y, Sassa Y, Hashizume H, Sekiguchi A, Fukushima A *et al.* Verbal working memory performance correlates with regional white matter structures in the fronto-parietal regions. *Neuropsychologia* 2011; **49**(12)**:** 3466-3473

12. Haier RJ, Jung RE, Yeo RA, Head K, Alkire MT. Structural brain variation and general intelligence. *Neuroimage* 2004; **23**(1)**:** 425-433.

13. Takeuchi H, Taki Y, Hashizume H, Sassa Y, Nagase T, Nouchi R *et al.* Failing to deactivate: the association between brain activity during a working memory task and creativity. *Neuroimage* 2011; **55**(2)**:** 681-687.

14. Takeuchi H, Taki Y, Thyreau B, Sassa Y, Hashizume H, Sekiguchi A *et al.* White matter structures associated with empathizing and systemizing in young adults. *Neuroimage* 2013; **77**(15)**:** 222-236.

15. Ashburner J, Friston KJ. Voxel-based morphometry-the methods. *Neuroimage* 2000; **11**(6)**:** 805-821.

16. Takeuchi H, Taki Y, Hashizume H, Asano K, Asano M, Sassa Y *et al.* The Impact of Television Viewing on Brain Structures: Cross-Sectional and Longitudinal Analyses. *Cereb Cortex* 2015; **25**(5)**:** 1188-1197.

17. Takeuchi H, Taki Y, Hashizume H, Sassa Y, Nagase T, Nouchi R *et al.* The association between resting functional connectivity and creativity. *Cereb Cortex* 2012; **22**(12)**:** 2921-2929.

18. Takeuchi H, Taki Y, Nouchi R, Hashizume H, Sassa Y, Sekiguchi A *et al.* Anatomical correlates of quality of life: Evidence from voxel-based morphometry. *Hum Brain Mapp* 2014; **35**(5)**:** 1834-1846.

19. Takeuchi H, Taki Y, Nouchi R, Hashizume H, Sekiguchi A, Kotozaki Y *et al.*

Anatomical correlates of self-handicapping tendency. *Cortex* 2013; **49**(4)**:** 1148-1154.

20. Takeuchi H, Taki Y, Nouchi R, Sekiguchi A, Kotozaki Y, Miyauchi C *et al.* Regional gray matter density is associated with achievement motivation: evidence from voxel-based morphometry. *Brain Struct Funct* 2014; **219**(1)**:** 71-83.

21. Takeuchi H, Taki Y, Sassa Y, Hashizume H, Sekiguchi A, Fukushima A *et al.* Brain structures associated with executive functions during everyday events in a non-clinical sample. *Brain Struct Funct* 2013; **218**(4)**:** 1017-1032.

22. Takeuchi H, Taki Y, Sassa Y, Hashizume H, Sekiguchi A, Nagase T *et al.* White matter structures associated with emotional intelligence: Evidence from diffusion tensor imaging. *Hum Brain Mapp* 2013; **34**(5)**:** 1025-1034.

23. Wallentin M. Putative sex differences in verbal abilities and language cortex: A critical review. *Brain Lang* 2009; **108**(3)**:** 175-183.

24. Silver M, Montana G, Nichols TE. False positives in neuroimaging genetics using voxel-based morphometry data. *Neuroimage* 2012; **54**(2)**:** 992-1000.

25. Kotozaki Y, Takeuchi H, Sekiguchi A, Araki T, Takahashi K, Yamamoto Y *et al.* POSITIVE EFFECTS OF THE VICTIM BY THE GROWING OF PLANTS

AFTER GREAT EAST JAPAN EARTHQUAKE. *International Journal of Recent Scientific Research* 2015; **6**(2)**:** 2850-2858.

26. Nakagawa S, Takeuchi H, Taki Y, Nouchi R, Sekiguchi A, Kotozaki Y *et al.* Comprehensive neural networks for guilty feelings in young adults. *Neuroimage* 2015; **105:** 248-256.

27. Kotozaki Y, Takeuchi H, Sekiguchi A, Yamamoto Y, Shinada T, Araki T *et al.* Biofeedback-based training for stress management in daily hassles: an intervention study. *Brain and behavior* 2014; **4**(4)**:** 566-579.

28. Kong F, Ding K, Yang Z, Dang X, Hu S, Song Y *et al.* Examining gray matter structures associated with individual differences in global life satisfaction in a large sample of young adults. *Soc Cogn Affect Neurosci* 2014**:** nsu144.

29. Yokoyama R, Nozawa T, Takeuchi H, Taki Y, Sekiguchi A, Nouchi R *et al.* Association between gray matter volume in the caudate nucleus and financial extravagance: findings from voxel-based morphometry. *Neurosci Lett* 2014; **563**(20)**:** 28-32.

30. Takeuchi H, Taki Y, Sassa Y, Hashizume H, Sekiguchi A, Fukushima A *et al.* Regional gray matter volume is associated with empathizing and systemizing in young adults. *PLoS ONE* 2014; **9**(1)**:** e84782.

31. Takeuchi H, Taki Y, Nouchi R, Sekiguchi A, Kotozaki Y, Miyauchi CM *et al.* A voxel-based morphometry study of gray and white matter correlates of a need for uniqueness. *Neuroimage* 2012; **63**(3)**:** 1119-1126.

32. Takeuchi H, Taki Y, Nouchi R, Hashizume H, Sekiguchi A, Kotozaki Y *et al.* The structure of the amygdala associates with human sexual permissiveness: Evidence from voxel-based morphometry. *Hum Brain Mapp* 2015; **36**(2)**:** 440-448.

33. Takeuchi H, Taki Y, Nouchi R, Hashizume H, Sekiguchi A, Kotozaki Y *et al.* Effects of Multitasking-Training on Gray Matter Structure and Resting State Neural Mechanisms. *Hum Brain Mapp* 2014; **35**(8)**:** 3646-3660.
